# Frailty in Aging HIV-Positive Individuals: An Evolving Healthcare Landscape

**DOI:** 10.7759/cureus.50539

**Published:** 2023-12-14

**Authors:** Mohammad Mansour, Monisha Augustine, Mahendra Kumar, Amna Naveed Butt, Thanmai Reddy Thugu, Parvinder Kaur, Nipakumari J Patel, Ankit Gaudani, M. Bilal Jahania, Elhama Jami, Mouhammad Sharifa, Rohan Raj, Dalia Mehmood

**Affiliations:** 1 General Medicine, University of Debrecen, Debrecen, HUN; 2 General Medicine, Jordan University Hospital, Amman, JOR; 3 Medicine, Kasturba Medical College, Manipal, Manipal, IND; 4 Medicine, Sardar Patel Medical College, Bikaner, Bikaner, IND; 5 Medicine/Internal Medicine, Allama Iqbal Medical College, Lahore, PAK; 6 Internal Medicine, Sri Padmavathi Medical College for Women, Sri Venkateswara Institute of Medical Sciences (SVIMS), Tirupati, IND; 7 Internal Medicine, Crimean State Medical University, Simferopol, UKR; 8 Internal Medicine, Soochow University, Suzhou, CHN; 9 Graduate Medical Education, Jiangsu University, Zhenjiang, CHN; 10 Internal Medicine, Combined Military Hospital (CMH) Lahore Medical College and Institute of Dentistry, Lahore, PAK; 11 Internal Medicine, Herat Regional Hospital, Herat, AFG; 12 Medicine, University of Aleppo, Faculty of Medicine, Aleppo, SYR; 13 Internal Medicine, Nalanda Medical College and Hospital, Patna, IND; 14 Community Medicine, Fatima Jinnah Medical University, Lahore, PAK

**Keywords:** geriatrics, frailty phenotype, clinical frailty scale, frailty screening, frailty index, treatment outcomes of hiv patients, old people living with hiv (oplwh), people living with hiv (plwh), hiv care, frailty

## Abstract

The life expectancy of people living with HIV (PLWH) has greatly increased due to advancements in combination antiretroviral treatment (cART). However, this longer life has also increased the prevalence of age-related comorbidities, such as frailty, which now manifest sooner in this group. Frailty, a term coined by the insurance industry, has been broadened to include physical, cognitive, and emotional elements and has been recognized as a critical predictor of negative health outcomes. With the median age of PLWH now in the mid-50s, treating frailty is critical given its link to chronic diseases, cognitive decline, and even death. Frailty assessment tools, such as the Frailty Phenotype (FP) and the Frailty Index (FI), are used to identify vulnerable people. Understanding the pathophysiology of frailty in PLWH indicates the role of immunological mechanisms. Frailty screening and management in this group have progressed, with specialized clinics and programs concentrating on multidisciplinary care. Potential pharmacotherapeutic solutions, as well as novel e-health programs and sensors, are in the future of frailty treatment, but it is critical to ensure that frailty evaluation is not exploited to perpetuate ageist healthcare practices. This narrative review investigates the changing healthcare environment for older people living with HIV (OPLWH), notably in high-income countries. It emphasizes the significance of identifying and managing frailty as a crucial feature of OPLWH's holistic care and well-being.

## Introduction and background

The development of more efficient, well-tolerated, and easy combination antiretroviral treatment (cART) has significantly enhanced HIV-positive people's (PLWH) life expectancy, even exceeding that of the general population [1]. Their extended survival, however, has resulted in a shift in their health profile, with a greater frequency of age-related comorbidities, which frequently manifest 5 to 10 years sooner than in the general population [2]. Furthermore, certain PLWH are becoming more susceptible to geriatric disorders such as frailty [3]. The goal of this study is to look into the role of frailty in the holistic treatment of older people with HIV (OPLWH).

Frailty, which was initially used by the insurance industry to assess mortality risks in adults over the age of 65 [4], has recently been broadened to include physical, cognitive, social, emotional, and economic components [5]. It denotes vulnerability to unfavorable health impacts as a result of several stresses. While frailty was originally studied in community-living persons over the age of 65, it has subsequently been studied across a wide range of demographics and conditions, indicating prevalence rates ranging from 3% to 5% between the ages of 30 and 60 to 25-30% after the age of 80 in the United Kingdom [6]. Frailty has been proven to be a more accurate predictor of survival in COVID-19 individuals than chronological age or comorbidities [7]. 

Frailty, cognitive decline, and other geriatric disorders are becoming more widespread and emerging sooner as PLWH continues to age, with a median age presently in the mid-50s in high-income countries [3]. Frailty must be identified since it increases the likelihood of acquiring new chronic diseases, such as falling, cognitive impairment, polypharmacy, hospitalization, loss of independence, and mortality. Because aging rates vary, screening for frailty is a critical initial step in the management of OPLWH [8].

As a result of these developments, the approach to caring for OPLWH has moved from immunovirologic treatment to a more multidisciplinary one that places an emphasis on tailored services [9]. Specialized programs have been developed to accommodate the particular needs of OPLWH [7]. While frailty assessments are not necessary for every OPLWH, they are crucial in identifying at-risk individuals since chronological age alone is not always a reliable predictor of age-related disorders in geriatrics [8].

## Review

Frailty: concept and evolution

Derived from the Latin word "fragilis" (meaning "breakable"), the term frailty indicates a condition of increased sensitivity. Fried devised the Frailty Phenotype (FP) and the Frailty Index (FI) to emphasize the complexity of the idea in contrast to impairments or comorbidities [10]. Although the fundamental traits and consequences of frailty are well acknowledged, the absence of a clear operational definition makes diagnosis difficult. There are at least 67 metrics that have been developed; the most often used ones in research are the FP and FI [11].

The FP diagnoses frailty when three of the five predefined physical criteria - unintentional weight loss, self-reported fatigue, poor hand grip strength, sluggish walking speed, and low physical activity - are met. One or two conditions indicate pre-frailty, whereas none indicate non-frailty. Based on the accumulation of many age-related health problems, the FI computes frailty. Generally speaking, frailty in older adults who live in communities is indicated by a FI greater than 0.25. A prognosis of poor quality and several acute illnesses is indicated by a FI larger than 0.7 [12]. There are 30 prerequisites in all. Even though the FP is simpler to use, it does require specialized assessments and qualified personnel. The FI, on the other hand, appears more complicated but provides superior risk discrimination and may be determined using electronic health information [13].

Models predict comparable results when applied to diverse populations, and they also identify subgroups of frail individuals who do not have impairments or comorbidities. Research on the prevalence of frailty varies, with estimates ranging from 2% to 25% [9]. Other proven methods for diagnosing frailty include the Edmonton Frail Scale (EFS), the Clinical Frailty Scale (CFS), and the Frail questionnaire. Even among those who are not yet diagnosed as frail, a meta-analysis confirmed the association between these markers of frailty and increased rates of death, hospitalization, loss of independence, impairments, falls, fractures, and cognitive decline [14].

A gradual loss of normal homeostatic mechanisms brought on by biological and environmental stresses that affect cells, tissues, organs, and the overall health of a person ultimately leads to frailty. A chronic inflammatory state is shaped by immunological traits and CMV seropositivity, and immunosenescence is a major factor in this process. Numerous processes and serum markers are associated with this chronic inflammation, which is sometimes referred to as inflammaging [15]. Hormonal fluctuations, epigenetic alterations, telomere shortening, genetic control, and changes in body composition, such as abdominal fat and muscle loss, can all have an impact on frailty [16].

The CFS is a measure of independence in older PLWH that shows favorable correlations with the FP and FI [17]. A multi-domain evaluation called the EFS has earlier been compared to the FP and FI in PLWH [18]. A simple instrument consisting of five parts is the FRAIL Scale [19]. Originally intended to predict death in PLWH, the Veterans Aging Cohort Study Index (VACS-I) is currently utilized as a frailty screening tool with construct validity and predictive validity [20]. These methods aid in identifying persons who, due to their frailty, may require treatments and care.

Pathophysiology of frailty in PLWH

Frailty in PLWH has molecular underpinnings associated with age-related dysregulation of several physiological systems. Dysregulated systems include those related to the stress response (neuro-immuno-endocrine), metabolism (insulin and mitochondria), and musculoskeletal systems [21]. When dysregulation escalates to a point where negative outcomes are more likely, frailty is categorized as a syndrome. Other variables are involved, even though the majority of evidence indicates that frailty in OPLWH is comparable to physiologic aging [22].

Prolonged low-grade HIV replication triggers persistent immunological activation even in suppressed individuals, which results in immunosenescence and inflammation that invariably produce frailty in clinical trials. Additionally, epigenomic dysregulation is at play. Clinical variables such as co-infections, early suppression of HIV replication, CD4/CD8 ratio, and microbiota translocation facilitate the development of frailty. These traits emphasize the role that immunoinflammatory variables play in the development of frailty [23]. Frailty is a common dysregulated condition of physiological functioning and impaired functional reserve that leads to negative outcomes typical of aging, although it may manifest earlier in PLWH [24].

Studies employing the FP for frailty diagnosis

In the multicenter AIDS Cohort Study (MACS), frailty was measured using an adapted frailty-related phenotype (aFRP) in untreated, seropositive, college-educated, Caucasian males with a mean age of 55. In the same group of males over 65 who tested negative for HIV, frailty was common in 3.4% of cases [25]. Immuno-virologic traits were associated with frailty, and among PLWH who started cART, frailty was associated with a higher risk of developing AIDS or passing away [26]. Risk factors for frailty included old age, non-Hispanic black ethnicity, and potentially controllable conditions such as AIDS, smoking, hepatitis C infection, depression, diabetes, and renal disease. Frailty also happens in the absence of concomitant comorbidities [25].

A 12% frailty prevalence was observed in the AIDS Linked to Intravenous Experience (ALIVE) cohort, which consisted primarily of male African-American injectable drug users (median age 49). Risk factors for this cohort included female sex, advanced HIV disease, lower education, depression, and multimorbidity, as well as older age and HIV infection. Frailty has been associated with all-cause hospitalizations [27], overall mortality [28], and chronic illnesses such as lung, heart, and mental disorders.

The HIV Infection, Aging, and Immune Function Long-Term Observational Study (HAILO) cohort examined disability and frailty. The prevalence of pre-frailty and frailty was 37% and 6% of the population, respectively, with little overlap between the two conditions. Premature mortality, type II diabetes, and cardiovascular disease have all been related to frailty [29]. Neurocognitive impairment, obesity, smoking, the first choice of cART (with NNRTI [nucleoside reverse transcriptase inhibitor]-based cART increasing the risk of frailty), and educational level were among the modifiable risk factors. Both moderate alcohol use and physical activity were protective [30].

Additionally, it was demonstrated that more seropositive women than males had frailty, which is in line with the overall population. 10.0% of uninfected women and 17.3% of seropositive women in the Women's Interagency HIV Study (WIHS) were frail [31]. Concerns regarding economic and healthcare delivery challenges as the PLWH population ages are raised by other research, like those carried out in South Africa and Spain, which revealed a high prevalence of frailty in PLWH [32].

Screening of frailty in PLWH

When a senior is diagnosed as frail, it signifies more than simply a condition with a dismal prognosis. For instance, one important risk factor for perioperative issues is frailty. By altering recognized preoperative variables for surgical morbidity, pre-habilitation clinics improve outcomes [33]. An integrated geriatric strategy is becoming more and more recommended for certain older PLWH, especially those who have been classified as weak [34].

Other surrogates, besides frailty, can help identify PLWH who would benefit from a geriatric assessment. For instance, polypharmacy is more common in PLWH compared to controls. Similarly, decreased functional status as determined by gait speed or the whole Short Physical Performance Battery (SPPB) assessment tool is also more common [35]. Frailty, functional status, and deficits all interact in PLWH, despite being distinct illnesses [36]. PLWH frequently experience functional impairments and impairments, especially those who also have concurrent geriatric symptoms [36,37]. Strong PLWH in need of geriatric review may also be indicated by a combination of immunological markers (e.g., a low nadir CD4 count of 200, a plateau CD4 level of 500 on suppressive cART, and a CD4/CD8 ratio of one) [38].

Weakness is a dynamic state. The majority of non-frail and pre-frail individuals retained their status in a 12-month follow-up analysis involving over 300 treated PLWH, but the majority of frail individuals relapsed to pre-frailty [39]. Pre-frailty, which affects 30-60% of PLWH, has to be identified since it is associated with unfavorable outcomes. We have previously discussed the elements that contribute to the shift of PLWH in the MACS to frailty. The only factor associated with a reversal of frailty was age [40]. Guaraldi spent four years studying the MHMC Cohort's frailty transition determinants. The following factors predicted FI at follow-up: baseline FI, female gender, length of HIV infection and cART usage, and history of smoking [41].

There are currently no guidelines for which PLWH to refer, and it is unclear if geriatric referrals are clinically effective. The public should be evaluated for frailty if they are older than 70. It is reasonable to think about screening PLWH above the age of 50 based on data showing PLWH age-advancement. The responsibilities of geriatricians as knowledgeable consultants or involved team members are being established [42].

Management of frailty in PLWH

In high-income nations, the primary healthcare approach for OPLWH is based on specialized community-based primary care or tertiary care that is offered in infectious disease clinics. Specialized geriatric or aging-HIV clinics, HIV-metabolic clinics, and HIV-rehabilitation programs have been established to address the wider range of social, emotional, and health concerns experienced by OPLWH as their healthcare needs have expanded to include non-HIV-related illnesses [43].

Numerous of these clinics have adopted care methods designed for senior citizens that are based on geriatric concepts. With an emphasis on modifying the Comprehensive Geriatric Assessment (CGA) to identify the most susceptible OPLWH, these models entail screening for frailty and other geriatric disorders. CGA, a well-researched multidimensional diagnostic procedure, is used to evaluate the functional, psychological, and medical abilities of a subset of elderly people [44]. After the age of 70, it is advised to do yearly frailty screenings in the general population [42]. According to recent guidelines, PLWH over 50 should undergo yearly frailty screening using the FRAIL Scale. If the screening findings are positive, the individual should then be sent for a thorough geriatric evaluation [45]. 

To test for frailty, HIV clinics that specialize in OPLWH use a variety of measures, such as gait speed, the FRAIL Scale, and the Clinical Frailty Scale [45]. Many clinics have provided insights into how they manage OPLWH who are frail, using multidisciplinary geriatric evaluations to determine who is most in need [34]. For instance, the CFS is used by the Chelsea and Westminster Clinic in London to screen older people with HIV (OPLWH). Individuals with a CFS score of 5 or more, which indicates at least mild frailty, are sent to a specialized geriatric HIV clinic [46]. Clinics may use several measures to screen for frailty. While some use electronic health records to identify the FP, others use the FI. When electronic health data are accessible, the FI may be applied more easily than the FP, which needs specific equipment and training [13, 47].

The goals of therapy for OPLWH who have been classified as frail are to prevent and control impairment and comorbidities. Furthermore, geriatric syndrome assessment and treatment are essential elements of this care approach [48]. To control OPLWH, tactics borrowed from research with frail, uninfected older adults are used. Maintaining the quality of life and possibly averting cognitive decline also depend on acknowledging the increased health risks linked to social isolation. This concept aims to actively involve older people with HIV/AIDS within their social networks and reintegrate HIV care into primary care while facilitating access to community resources [49].

Fundamental care principles for frail older HIV-negative individuals include implementing strength-training exercise programs, managing sarcopenia, assessing for polypharmacy, treating weight loss and undernutrition with protein-calorie supplements, and evaluating and treating reversible causes of fatigue such as anemia, depression, hypothyroidism, and B12 deficiency. Furthermore, vitamin D levels should be monitored, and it should be administered if necessary [50,51].

Programs for preventing frailty that involve many components have shown promise in reducing the pace of frailty development, boosting cognitive performance, and increasing physical function [52]. CGA informs care plans that can improve functional abilities, lower the chance of institutionalization, postpone the onset of impairment, cut down on hospital admissions and stays, and increase survival [53]. A physiotherapist should ideally be available or included in the multidisciplinary team. Exercise programs of varying intensities have enhanced physical function in OPLWH, while short-duration exercise programs have shown beneficial effects on frail older individuals. Improvements in quality-of-life measures in PLWH have also been associated with participation in yoga programs [54].

While exercise has been shown to be the most effective intervention for frailty, group-based physiotherapy classes have also demonstrated efficacy in protecting against functional decline. On the other hand, addressing the social determinants of frailty is important. This includes, but is not limited to, isolation, loneliness, depression, or any other psychiatric diagnoses. However, this may not always be feasible, as it often depends on the availability of local professional resources and the presence of a community-based support system [55].

The New Orleans Alcohol Use in HIV (NOAH) study evaluated the body composition, gait speed, and muscle strength of 341 participants living with HIV. Body composition was found to have a modulatory effect on frailty risk among PLWH, which was statistically significant. That is, while obesity was associated with increased risk, greater muscle mass may have had a protective effect, even among individuals who consume alcohol. These findings emphasize the importance of physical activity and weight control in modulating frailty risk [56].

Furthermore, strict control of the medications PLWH consumes may be beneficial. Polypharmacy may increase the risk of adverse outcomes, and de-prescription is often necessary. For instance, a study demonstrated that frailty was more common in those who used medications with an anticholinergic effect (OR 2.12; 95% CI 0.89-5.0). Therefore, clinicians should be aware of their impact and work to reduce exposure whenever possible [57].

As mentioned earlier, exercise is a well-established preventive intervention for frailty. However, since many of the OPLWH are already in a frail state, high-intensity training and weight-lifting may be too taxing. Therefore, a study investigated a novel game-based training program, referred to as exergame, and its impact on ameliorating frailty among 10 HIV-infected individuals. The exergame program which was conducted twice weekly for six weeks, incorporated activities such as weight shifting, ankle reaching, and obstacle crossing. After the conclusion of the program, changes in balance, gait, and other parameters were assessed, and improvements were seen in balance and mobility [58].

E-health systems are being increasingly utilized to provide health information, personalized recommendations, and smartphone reminders to promote healthy habits and help older people preserve and enhance their functional independence. There is much promise in the continuing evaluation of wearable sensors for frailty identification [54]. The Ecological Momentary Assessment, which gathers real-time patient-reported outcome indicators, can help make it easier to incorporate patient-reported outcomes into innovative healthcare models [53]. Nonetheless, it is important to maintain vigilance to prevent the possible ageist approach to healthcare delivery from being supported by frailty [52].

Limitations and challenges

While our review provides insight into frailty pathophysiology, screening, detection, and management for PLWH, it also identifies several limitations and research gaps, the most notable of which is the absence of a standardized operational definition for frailty. This presents a challenge not only for consistent diagnosis in the healthcare setting but also for conducting studies with reliable data and results. Therefore, the need to establish a universally accepted definition is paramount. However, more research is needed to explore the most effective and relevant metrics for frailty while considering the diverse impacted populations, including OPLWH.

In a comparable context, the variability of estimates of frailty prevalence in the different studies (ranging from 2% to 25%) highlights the importance of understanding any contributing factors and standardizing assessment methods in future studies. On the other hand, it implies that frailty in this population (PLWH) may manifest differently. Furthermore, while our article touches on the integration of geriatric concepts in HIV care, more research is required to assess the feasibility, effectiveness, and scalability of these approaches. Clear guidelines for identifying PLWH who would benefit from frailty assessments and evaluating the clinical effectiveness of geriatric referrals are lacking, and this is an area we aim to address in our ongoing research.

Lastly, a more thorough exploration of e-health systems and electronic health records used for estimating frailty, especially those employing metrics such as the Frailty Index, would enhance our understanding of technological advancements in frailty assessment and their real-world applicability. Addressing these gaps will contribute to a more comprehensive and nuanced understanding of frailty, improve diagnostic accuracy and inform targeted interventions for vulnerable populations.

## Conclusions

As OPLWH lives longer, healthcare for them is changing. However, there are issues with the growth in frailty and other geriatric problems among OPLWH. Once associated with elderly people, fragility is now a critical factor in determining susceptibility to unfavorable health outcomes. Personalized services and an interdisciplinary approach have been developed for OPLWH. Because frailty is associated with mortality and chronic diseases, it is imperative to identify and treat it early. Research on pharmacotherapeutic alternatives and e-health solutions is still ongoing. To offer complete treatment in this evolving healthcare environment, it is vital to steer clear of ageist healthcare practices and give frailty the attention it demands.
